# The genome sequence of Fabricius’ Nomad Bee, *Nomada fabriciana *(Linne, 1767)

**DOI:** 10.12688/wellcomeopenres.20184.1

**Published:** 2023-10-26

**Authors:** Liam M. Crowley, University of Oxford and Wytham Woods Genome Acquisition Lab, Darwin Tree of Life Barcoding collective, Wellcome Sanger Institute Tree of Life programme, Wellcome Sanger Institute Scientific Operations: DNA Pipelines collective, Tree of Life Core Informatics collective, Darwin Tree of Life Consortium

**Affiliations:** 1 Department of Biology, University of Oxford, Oxford, England, UK

**Keywords:** Nomada fabriciana, Fabricius’ Nomad Bee, genome sequence, chromosomal, Hymenoptera

## Abstract

We present a genome assembly from an individual female *Nomada fabriciana* (Fabricius’ Nomad Bee; Arthropoda; Insecta; Hymenoptera; Apidae). The genome sequence is 233.6 megabases in span. Most of the assembly is scaffolded into 12 chromosomal pseudomolecules. The mitochondrial genome has also been assembled and is 19.4 kilobases in length. Gene annotation of this assembly on Ensembl identified 9,700 protein coding genes.

## Species taxonomy

Eukaryota; Metazoa; Eumetazoa; Bilateria; Protostomia; Ecdysozoa; Panarthropoda; Arthropoda; Mandibulata; Pancrustacea; Hexapoda; Insecta; Dicondylia; Pterygota; Neoptera; Endopterygota; Hymenoptera; Apocrita; Aculeata; Apoidea; Anthophila; Apidae; Nomadinae; Nomadini; *Nomada*; *Nomada fabriciana* (Linne, 1767) (NCBI:txid601510).

## Background

Fabricius’ Nomad Bee, *Nomada fabriciana*, is a small nomad bee in the family Apidae. It occurs across the Palaearctic and is common and widespread across the south of the UK, becoming scarcer further north with few records in Scotland and Ireland. *Nomada* are relatively hairless bees, causing possible confusion with wasps for the inexperienced. Identification of *Nomada* can be challenging, although *N. fabriciana* is easily recognised by its small size, dark overall appearance, reddish gaster and the unique combination of a black labrum and bidentate mandibles. Furthermore, females have antennae with a red base and tip with a band of black segments in the middle. The typical form has yellow spots on tergite two, although dark forms occur which lack this along with almost entirely dark legs and antennae.

*Nomada* are brood parasites of other bees, mainly attacking Andrena although some species are kleptoparasites of other bee genera ( Odanaka *et al.*, 2022). Female Nomad bees can often be seen patrolling and investigating nesting sites of the host. They enter the host nest and lay their own eggs on the wall of unsealed cells. The larvae hatch and proceed to kill the host egg or larva using large, well-developed mandibles, before consuming the pollen provisions ( Rozen Jr, 1991). The main host of *N. fabriciana* is *Andrena bicolor*, although other hosts are used including *A. angustior*, *A. chrysosceles*, *A. flavipes* and *A. nigroaenea* ( Falk & Lewington, 2019). It is likely that the large size variation in the species is determined by the size of the host.

It is associated with a large range of habitats and can be found anywhere that its hosts occur. In the UK it is largely bivoltine with the first flight period from March to June, and the second from June to August ( Else & Edwards, 2018). Adults visit a wide range of flowers for nectar, including dandelion ( *Taraxacum* sp.), daisy ( *Bellis perennis*), ragwort ( *Senecio jacobaea*), field scabious ( *Knautia arvensis*), speedwell ( *Veronica* spp.), spurge ( *Euphorbia* spp.), stitchwort ( *Stellaria* spp.), strawberry ( *Fagaria vesca*) and willow ( *Salix* spp.) ( Else & Edwards, 2018).

This complete genome represents one of the first for the genus, which has important and biological differences to other genera within the Apidae that includes ecologically and economically important species, such as the Honeybee ( *Apis mellifera*) and bumblebees ( *Bombus* spp.). As such, this genome will facilitate studies into research areas such as the evolution of kleptoparasitism, sociality and reproductive systems and well as providing useful data for resolving Hymenopteran taxonomy.

## Genome sequence report

**Figure 1.**
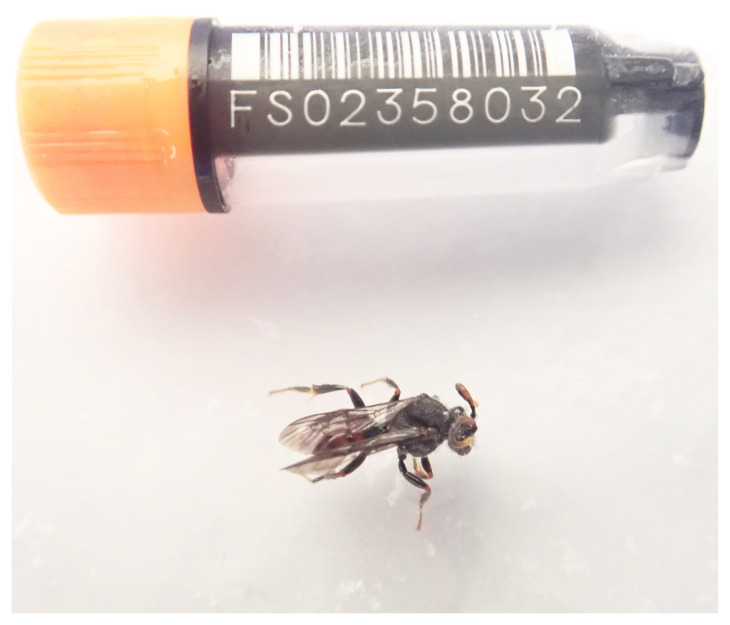
Photograph of the *Nomada fabriciana* (iyNomFabr1) specimen used for genome sequencing.

The genome was sequenced from one female *Nomada fabriciana* ( Figure 1) collected from Wytham Woods, Oxfordshire, UK (51.77, –1.34). A total of 99-fold coverage in Pacific Biosciences single-molecule HiFi long reads and 154-fold coverage in 10X Genomics read clouds were generated. Primary assembly contigs were scaffolded with chromosome conformation Hi-C data. Manual assembly curation corrected 11 missing joins or mis-joins, reducing the scaffold number by 3.5%, and increasing the scaffold N50 by 22.04%.

**Figure 2.**
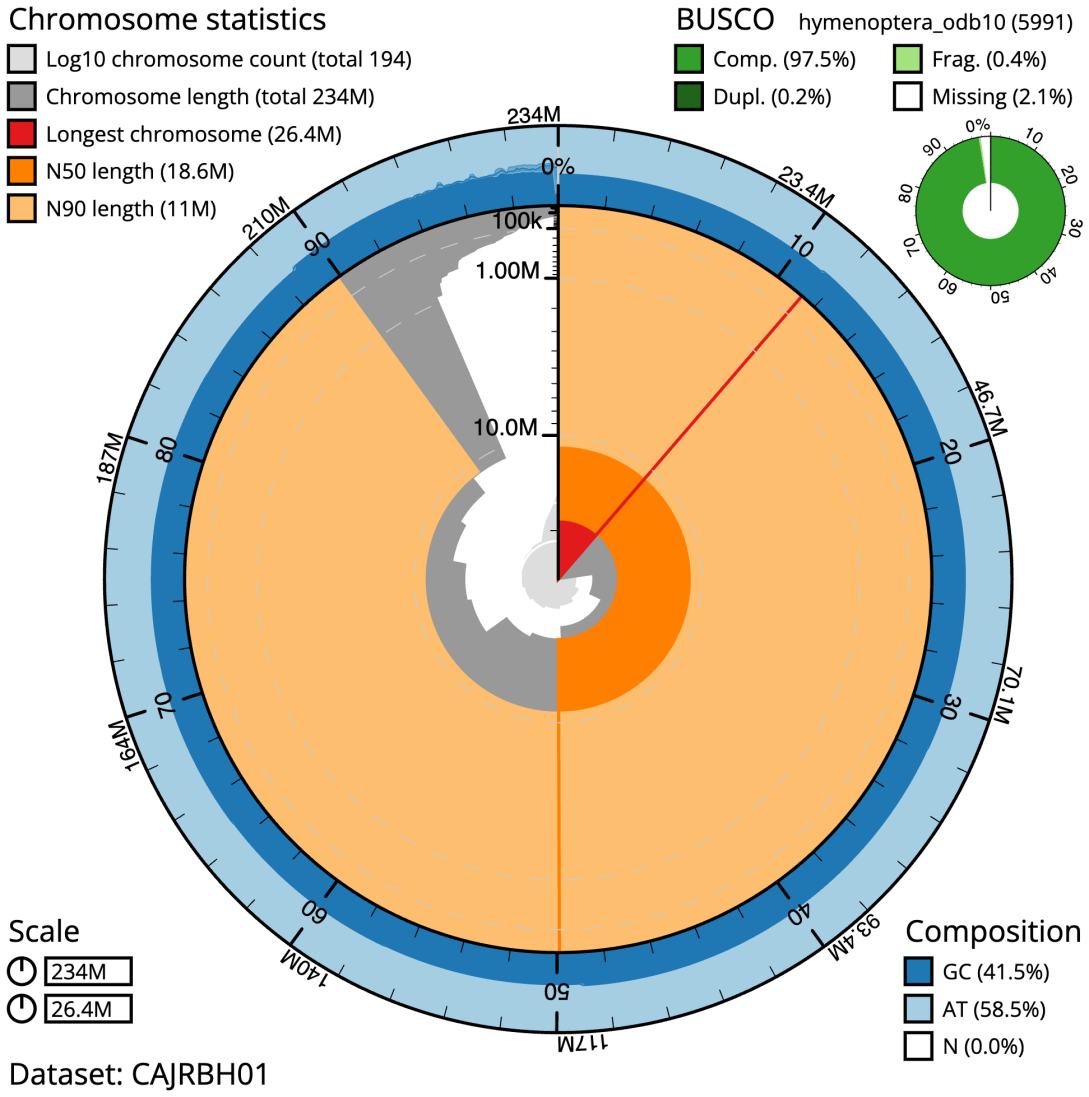
Genome assembly of *Nomada fabriciana*, iyNomFabr1.1: metrics. The BlobToolKit Snailplot shows N50 metrics and BUSCO gene completeness. The main plot is divided into 1,000 size-ordered bins around the circumference with each bin representing 0.1% of the 233,603,770 bp assembly. The distribution of scaffold lengths is shown in dark grey with the plot radius scaled to the longest scaffold present in the assembly (26,420,868 bp, shown in red). Orange and pale-orange arcs show the N50 and N90 scaffold lengths (18,567,632 and 10,996,985 bp), respectively. The pale grey spiral shows the cumulative scaffold count on a log scale with white scale lines showing successive orders of magnitude. The blue and pale-blue area around the outside of the plot shows the distribution of GC, AT and N percentages in the same bins as the inner plot. A summary of complete, fragmented, duplicated and missing BUSCO genes in the hymenoptera_odb10 set is shown in the top right. An interactive version of this figure is available at https://blobtoolkit.genomehubs.org/view/Nomada%20fabriciana/dataset/CAJRBH01/snail.

**Figure 3.**
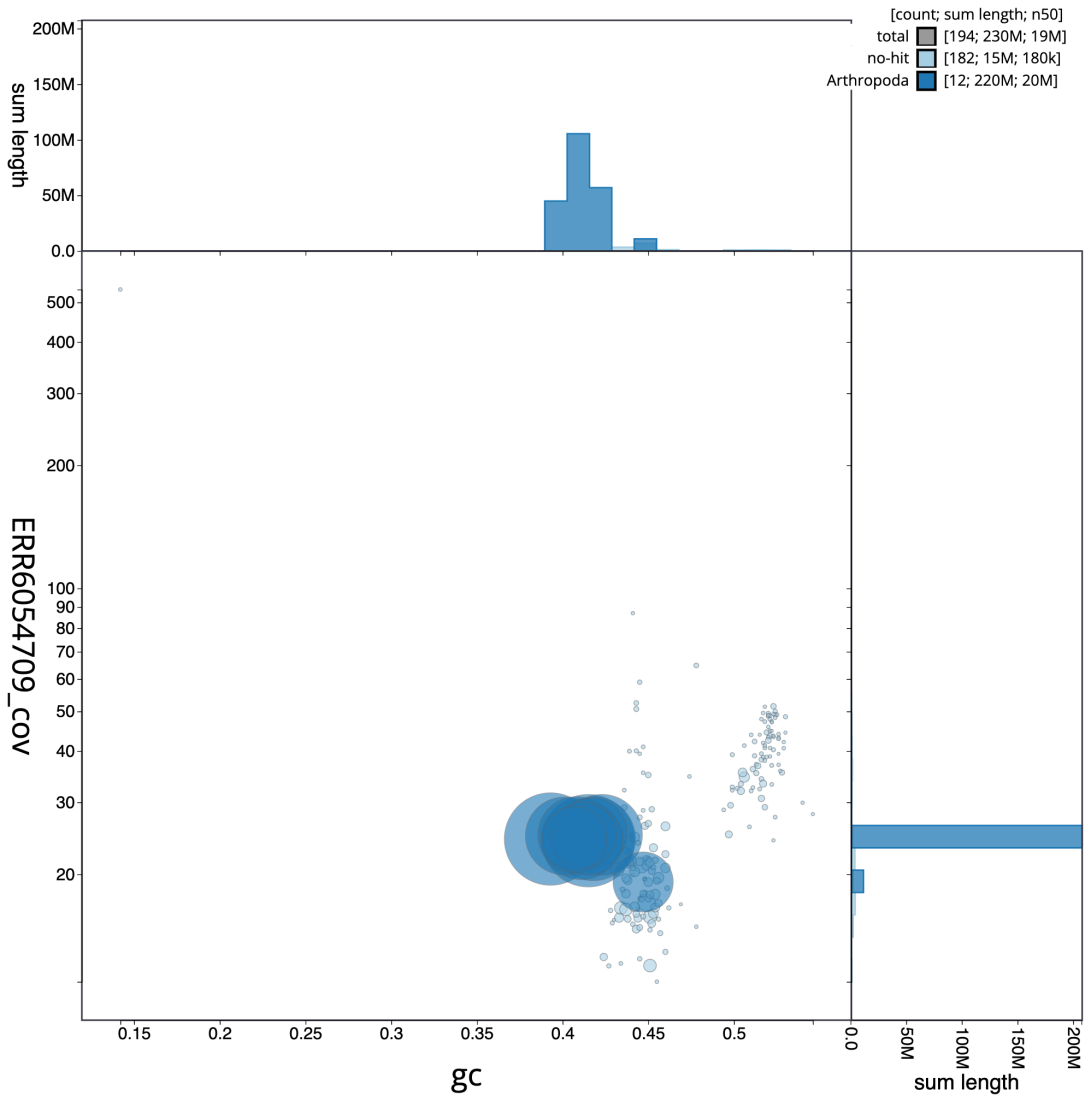
Genome assembly of *Nomada fabriciana*, iyNomFabr1.1: BlobToolKit GC-coverage plot. Scaffolds are coloured by phylum. Circles are sized in proportion to scaffold length. Histograms show the distribution of scaffold length sum along each axis. An interactive version of this figure is available at https://blobtoolkit.genomehubs.org/view/Nomada%20fabriciana/dataset/CAJRBH01/blob.

**Figure 4.**
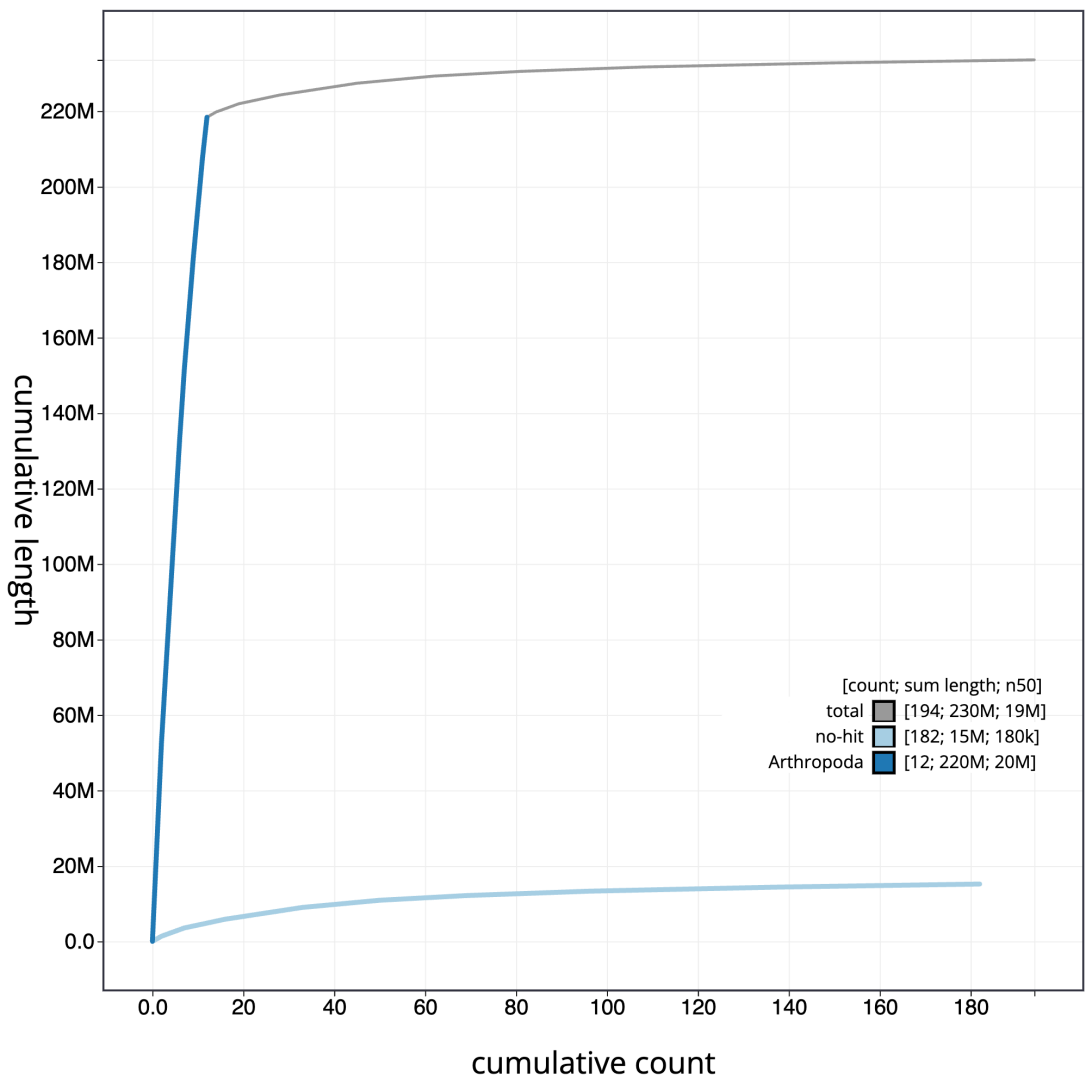
Genome assembly of *Nomada fabriciana*, iyNomFabr1.1: BlobToolKit cumulative sequence plot. The grey line shows cumulative length for all scaffolds. Coloured lines show cumulative lengths of scaffolds assigned to each phylum using the buscogenes taxrule. An interactive version of this figure is available at https://blobtoolkit.genomehubs.org/view/Nomada%20fabriciana/dataset/CAJRBH01/cumulative.

**Figure 5.**
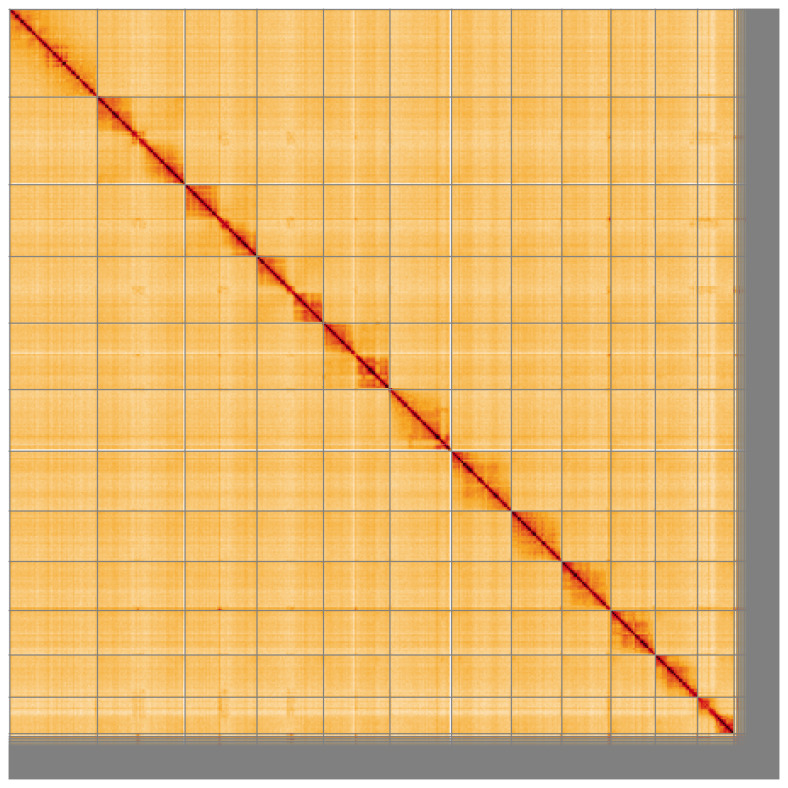
Genome assembly of *Nomada fabriciana*, iyNomFabr1.1: Hi-C contact map of the iyNomFabr1.1 assembly, visualised using HiGlass. Chromosomes are shown in order of size from left to right and top to bottom. An interactive version of this figure may be viewed at https://genome-note-higlass.tol.sanger.ac.uk/l/?d=Xd136OeBQwyuHs1mVJaVWg.

The final assembly has a total length of 233.6 Mb in 193 sequence scaffolds with a scaffold N50 of 18.6 Mb ( Table 1). A summary of the assembly statistics is shown in Figure 2, while the distribution of assembly scaffolds on GC proportion and coverage is shown in Figure 3. The cumulative assembly plot in Figure 4 shows curves for subsets of scaffolds assigned to different phyla. Most (95.24%) of the assembly sequence was assigned to 12 chromosomal-level scaffolds. Chromosome-scale scaffolds confirmed by the Hi-C data are named in order of size ( Figure 5; Table 2). The specimen is a diploid female. While not fully phased, the assembly deposited is of one haplotype. Contigs corresponding to the second haplotype have also been deposited. The mitochondrial genome was also assembled and can be found as a contig within the multifasta file of the genome submission.

**Table 1.** Genome data for *Nomada fabriciana*, iyNomFabr1.1.

Project accession data		
Assembly identifier	iyNomFabr1.1	
Assembly release date	2021-05-17	
Species	*Nomada fabriciana*	
Specimen	iyNomFabr1	
NCBI taxonomy ID	601510	
BioProject	PRJEB44833	
BioSample ID	SAMEA7520701	
Isolate information	iyNomFabr1, female: whole organism (DNA sequencing and Hi-C data)	
Assembly metrics *		*Benchmark*
Consensus quality (QV)	53.8	*≥ 50*
*k*-mer completeness	99.99%	*≥ 95%*
BUSCO **	C:97.5%[S:97.3%,D:0.2%],F:0.4%,M:2.1%,n:5,991	*C ≥ 95%*
Percentage of assembly mapped to chromosomes	95.24%	*≥ 95%*
Sex chromosomes	-	*localised homologous pairs*
Organelles	Mitochondrial genome assembled	*complete single alleles*
Raw data accessions		
PacificBiosciences SEQUEL II	ERR6436371	
10X Genomics Illumina	ERR6054710, ERR6054707, ERR6054709, ERR6054708	
Hi-C Illumina	ERR6054711	
Genome assembly		
Assembly accession	GCA_907165295.1	
*Accession of alternate haplotype*	GCA_907165305.1	
Span (Mb)	233.6	
Number of contigs	224	
Contig N50 length (Mb)	13.4	
Number of scaffolds	193	
Scaffold N50 length (Mb)	18.6	
Longest scaffold (Mb)	26.4	
Genome annotation		
Number of protein-coding genes	9,700	
Number of non-coding genes	1,659	
Number of gene transcripts	17,002	

* Assembly metric benchmarks are adapted from column VGP-2020 of “Table 1: Proposed standards and metrics for defining genome assembly quality” from ( Rhie *et al.*, 2021). ** BUSCO scores based on the hymenoptera_odb10 BUSCO set using v5.3.2. C = complete [S = single copy, D = duplicated], F = fragmented, M = missing, n = number of orthologues in comparison. A full set of BUSCO scores is available at https://blobtoolkit.genomehubs.org/view/Nomada%20fabriciana/dataset/CAJRBH01/busco.

**Table 2.** Chromosomal pseudomolecules in the genome assembly of *Nomada fabriciana*, iyNomFabr1.

INSDC accession	Chromosome	Length (Mb)	GC%
OU015688.1	1	26.42	39.5
OU015689.1	2	26.41	41.5
OU015690.1	3	21.68	42.0
OU015691.1	4	20.07	41.0
OU015692.1	5	20.05	42.5
OU015693.1	6	18.57	40.0
OU015694.1	7	18.04	41.0
OU015695.1	8	15.21	41.5
OU015696.1	9	14.8	41.5
OU015697.1	11	13.4	41.0
OU015698.1	12	12.76	40.5
OU015699.1	10	11.0	44.5
OU015700.1	MT	0.02	14.5

The estimated Quality Value (QV) of the final assembly is 53.8 with *k*-mer completeness of 99.99%, and the assembly has a BUSCO v5.3.2 completeness of 97.5% (single = 97.3%, duplicated = 0.2%), using the hymenoptera_odb10 reference set ( *n* = 5,911).

Metadata for specimens, spectral estimates, sequencing runs, contaminants and pre-curation assembly statistics can be found at https://links.tol.sanger.ac.uk/species/601510.

## Genome annotation report

The *Nomada fabriciana* genome assembly (GCA_907165295.1) was annotated using the Ensembl rapid annotation pipeline ( Table 1; https://rapid.ensembl.org/Nomada_fabriciana_GCA_907165295.1/Info/Index). The resulting annotation includes 17,002 transcribed mRNAs from 9,700 protein-coding and 1,659 non-coding genes.

## Methods

### Sample acquisition and nucleic acid extraction

A female *Nomada fabriciana* (specimen ID Ox000487, ToLID iyNomFabr1) was netted in Wytham Woods, Oxfordshire (biological vice-county Berkshire), UK (latitude 51.77, longitude –1.34) on 2020-06-15. The specimen was collected and identified by Liam Crowley (University of Oxford) and preserved on dry ice.

DNA was extracted at the Tree of Life laboratory, Wellcome Sanger Institute (WSI). The iyNomFabr1 sample was weighed and dissected on dry ice with tissue set aside for Hi-C sequencing. Tissue from the whole organism was disrupted using a Nippi Powermasher fitted with a BioMasher pestle. High molecular weight (HMW) DNA was extracted using the Qiagen MagAttract HMW DNA extraction kit. Low molecular weight DNA was removed from a 20 ng aliquot of extracted DNA using the 0.8X AMpure XP purification kit prior to 10X Chromium sequencing; a minimum of 50 ng DNA was submitted for 10X sequencing. HMW DNA was sheared into an average fragment size of 12–20 kb in a Megaruptor 3 system with speed setting 30. Sheared DNA was purified by solid-phase reversible immobilisation using AMPure PB beads with a 1.8X ratio of beads to sample to remove the shorter fragments and concentrate the DNA sample. The concentration of the sheared and purified DNA was assessed using a Nanodrop spectrophotometer and Qubit Fluorometer and Qubit dsDNA High Sensitivity Assay kit. Fragment size distribution was evaluated by running the sample on the FemtoPulse system.

### Sequencing

Pacific Biosciences HiFi circular consensus and 10X Genomics read cloud DNA sequencing libraries were constructed according to the manufacturers’ instructions. DNA sequencing was performed by the Scientific Operations core at the WSI on Pacific Biosciences SEQUEL II (HiFi) and HiSeq X Ten (10X) instruments. Hi-C data were also generated from remaining tissue of iyNomFabr1 using the Arima2 kit and sequenced on the HiSeq X Ten instrument.

### Genome assembly, curation and evaluation

Assembly was carried out with Hifiasm ( Cheng *et al.*, 2021) and haplotypic duplication was identified and removed with purge_dups ( Guan *et al.*, 2020). One round of polishing was performed by aligning 10X Genomics read data to the assembly with Long Ranger ALIGN, calling variants with FreeBayes ( Garrison & Marth, 2012). The assembly was then scaffolded with Hi-C data ( Rao *et al.*, 2014) using SALSA2 ( Ghurye *et al.*, 2019). The assembly was checked for contamination and corrected using the gEVAL system ( Chow *et al.*, 2016) as described previously ( Howe *et al.*, 2021). Manual curation was performed using gEVAL, HiGlass ( Kerpedjiev *et al.*, 2018) and Pretext ( Harry, 2022). The mitochondrial genome was assembled using MitoHiFi ( Uliano-Silva *et al.*, 2023), which runs MitoFinder ( Allio *et al.*, 2020) or MITOS ( Bernt *et al.*, 2013) and uses these annotations to select the final mitochondrial contig and to ensure the general quality of the sequence.

A Hi-C map for the final assembly was produced using bwa-mem2 ( Vasimuddin *et al.*, 2019) in the Cooler file format ( Abdennur & Mirny, 2020). To assess the assembly metrics, the *k*-mer completeness and QV consensus quality values were calculated in Merqury ( Rhie *et al.*, 2020). This work was done using Nextflow ( Di Tommaso *et al.*, 2017) DSL2 pipelines “sanger-tol/readmapping” ( Surana *et al.*, 2023a) and “sanger-tol/genomenote” ( Surana *et al.*, 2023b). The genome was analysed within the BlobToolKit environment ( Challis *et al.*, 2020) and BUSCO scores ( Manni *et al.*, 2021; Simão *et al.*, 2015) were calculated.

Table 3 contains a list of relevant software tool versions and sources.

**Table 3.** Software tools: versions and sources.

Software tool	Version	Source
BlobToolKit	4.1.7	https://github.com/blobtoolkit/blobtoolkit
BUSCO	5.3.2	https://gitlab.com/ezlab/busco
FreeBayes	1.3.1-17-gaa2ace8	https://github.com/freebayes/freebayes
gEVAL	N/A	https://geval.org.uk/
Hifiasm	0.12	https://github.com/chhylp123/hifiasm
HiGlass	1.11.6	https://github.com/higlass/higlass
Long Ranger ALIGN	2.2.2	https://support.10xgenomics.com/genome-exome/software/pipelines/latest/advanced/other-pipelines
Merqury	MerquryFK	https://github.com/thegenemyers/MERQURY.FK
MitoHiFi	2	https://github.com/marcelauliano/MitoHiFi
PretextView	0.2	https://github.com/wtsi-hpag/PretextView
purge_dups	1.2.3	https://github.com/dfguan/purge_dups
SALSA	2.2	https://github.com/salsa-rs/salsa
sanger-tol/genomenote	v1.0	https://github.com/sanger-tol/genomenote
sanger-tol/readmapping	1.1.0	https://github.com/sanger-tol/readmapping/tree/1.1.0

### Genome annotation

The Ensembl gene annotation system ( Aken *et al.*, 2016) was used to generate annotation for the *Nomada fabriciana* assembly (GCA_907165295.1). Annotation was created primarily through alignment of transcriptomic data to the genome, with gap filling via protein-to-genome alignments of a select set of proteins from UniProt ( UniProt Consortium, 2019).

### Wellcome Sanger Institute – Legal and Governance

The materials that have contributed to this genome note have been supplied by a Darwin Tree of Life Partner. The submission of materials by a Darwin Tree of Life Partner is subject to the **‘Darwin Tree of Life Project Sampling Code of Practice’**, which can be found in full on the Darwin Tree of Life website here. By agreeing with and signing up to the Sampling Code of Practice, the Darwin Tree of Life Partner agrees they will meet the legal and ethical requirements and standards set out within this document in respect of all samples acquired for, and supplied to, the Darwin Tree of Life Project. 

Further, the Wellcome Sanger Institute employs a process whereby due diligence is carried out proportionate to the nature of the materials themselves, and the circumstances under which they have been/are to be collected and provided for use. The purpose of this is to address and mitigate any potential legal and/or ethical implications of receipt and use of the materials as part of the research project, and to ensure that in doing so we align with best practice wherever possible. The overarching areas of consideration are:

•   Ethical review of provenance and sourcing of the material

•   Legality of collection, transfer and use (national and international)

Each transfer of samples is further undertaken according to a Research Collaboration Agreement or Material Transfer Agreement entered into by the Darwin Tree of Life Partner, Genome Research Limited (operating as the Wellcome Sanger Institute), and in some circumstances other Darwin Tree of Life collaborators.

## Data Availability

European Nucleotide Archive: *Nomada fabriciana* (Fabricius’ nomad bee). Accession number PRJEB44833; https://identifiers.org/ena.embl/PRJEB44833. ( Wellcome Sanger Institute, 2021) The genome sequence is released openly for reuse. The *Nomada fabriciana* genome sequencing initiative is part of the Darwin Tree of Life (DToL) project. All raw sequence data and the assembly have been deposited in INSDC databases. Raw data and assembly accession identifiers are reported in Table 1.

## Author information

Members of the University of Oxford and Wytham Woods Genome Acquisition Lab are listed here: https://doi.org/10.5281/zenodo.4789928.

Members of the Darwin Tree of Life Barcoding collective are listed here: https://doi.org/10.5281/zenodo.4893703.

Members of the Wellcome Sanger Institute Tree of Life programme are listed here: https://doi.org/10.5281/zenodo.4783585.

Members of Wellcome Sanger Institute Scientific Operations: DNA Pipelines collective are listed here: https://doi.org/10.5281/zenodo.4790455.

Members of the Tree of Life Core Informatics collective are listed here: https://doi.org/10.5281/zenodo.5013541.

Members of the Darwin Tree of Life Consortium are listed here: https://doi.org/10.5281/zenodo.4783558.
