# Supplementary material for: The genome sequence of Fabricius’ Nomad Bee, Nomada fabriciana (Linne, 1767)
Source: Wellcome Open Res. 2023 Oct 26;8:497. [Version 1] doi: 10.12688/wellcomeopenres.20184.1 (PMC10980859; doi:10.12688/wellcomeopenres.20184.1)
Supplement: Peer review history

# Reviewer report: Reviewer response for version 1

**DOI:** 10.21956/wellcomeopenres.22347.r75158

**Published:** 2024-03-29

**Authors:** Ryan Brock

**Affiliations:** 1 Department of Crop Genetics, John Innes Centre, Norwich Research Park, Norwich, UK

This report outlines the genome assembly and annotation of Fabricius’ Nomad Bee ( *Nomada fabriciana*). The Background section provides a brief natural history overview (description, range, life cycle, and foraging preferences) for *N. fabriciana *and underlines how a genome for this species is important for understanding social evolution and the evolution of kleptoparasitic life-histories. More generally, the genome will also be of use in understanding pollinator genetic diversity and Hymenopteran taxonomy.

 All methods used are appropriate for invertebrate genome assembly and result in a high-quality and well-annotated genome that is clearly presented with interactive figures and tables throughout the report. Importantly, the methods for every step (sample acquisition, DNA extraction, sequencing, assembly, and annotation) are provided in detail, allowing replication of each step by other researchers if necessary. The software versions for all bioinformatic tools, along with links to protocols and pipelines for each assembly and annotation step are provided, improving reproducibility. This is something that I think these genome reports have fallen short on in the past, so it’s good to see this addressed.

 Finally, all datasets generated are presented in an easily accessible manner, allowing other researchers to utilise these genomic resources. It’s great to see that the raw read data and pre-curation assembly statistics are now linked within these reports, which further improves transparency and reproducibility.

Are sufficient details of methods and materials provided to allow replication by others?

Yes

Is the rationale for creating the dataset(s) clearly described?

Yes

Are the datasets clearly presented in a useable and accessible format?

Yes

Are the protocols appropriate and is the work technically sound?

Yes

Reviewer Expertise:

insect evolution; population genetics; behavioural ecology; plant-insect interactions

I confirm that I have read this submission and believe that I have an appropriate level of expertise to confirm that it is of an acceptable scientific standard.

# Reviewer report: Reviewer response for version 1

**DOI:** 10.21956/wellcomeopenres.22347.r75149

**Published:** 2024-03-14

**Authors:** Luciano Palmieri

**Affiliations:** 1 Faculty of Agricultural, Free University of Bolzen, Bolzano State, Bolzano, 39100, Italy; 2 Department of Integrative Biology, University of Wisconsin-Madison, Madison, Wisconsin, USA

The manuscript presents a clear rationale for creating the datasets associated with *Nomada fabriciana*. It elucidates the significance of understanding the genomic composition of this species, highlighting its importance in studying evolutionary dynamics, social behavior, and taxonomy within the Apidae family. The authors underscore the biological and ecological relevance of *Nomada fabriciana*, particularly in relation to its role as a brood parasite and its interactions with host species.

 The methods described in the manuscript are appropriate for the objectives of the study. The authors clearly describe the sample acquisition process, DNA extraction, and sequencing, including the use of PacBio single-molecule HiFi long reads, 10X Genomics read clouds, and Hi-C data for assembly and scaffolding. The integration of multiple sequencing technologies and manual curation steps enhances the accuracy and completeness of the genomic dataset.

 The manuscript also provides sufficient details of methods and materials, enabling replication by others in the scientific community. It delineates the procedures for sample collection, DNA extraction, library preparation, and sequencing, along with software tools and pipelines utilized for genome assembly, curation, and annotation. The inclusion of specific laboratory protocols, sequencing parameters, and software versions enhances the transparency and reproducibility of the research findings.

 The datasets are presented in a usable and accessible format, facilitating further analysis and exploration by other researchers. The assembly statistics, genome annotation results, and quality metrics are clearly presented, providing comprehensive insights into the genomic characteristics of *Nomada fabriciana*. Additionally, he deposition of genomic data in public repositories ensures broad accessibility and promotes collaboration within the scientific community. The authors' thorough approach to experimental design, execution, and data dissemination contributes to the advancement of knowledge in bee biology and genomics.

Are sufficient details of methods and materials provided to allow replication by others?

Yes

Is the rationale for creating the dataset(s) clearly described?

Yes

Are the datasets clearly presented in a useable and accessible format?

Yes

Are the protocols appropriate and is the work technically sound?

Yes

Reviewer Expertise:

Coleoptera, Systematics, Biogeography, Biodiversity, Taxonomy, Entomology, Insect Taxonomy, Evolution,  Natural History,  Molecular Phylogenetics, Invertebrate Zoology, Parasitoids, Macroevolution, Genome Assembly and Phylogenomics.

I confirm that I have read this submission and believe that I have an appropriate level of expertise to confirm that it is of an acceptable scientific standard.
